# Mechanisms of parasite‐mediated disruption of brain vessels

**DOI:** 10.1002/1873-3468.70255

**Published:** 2025-12-18

**Authors:** Leonor Loira, Sílvia Arroz‐Madeira, Cláudio A. Franco, Sara Silva Pereira

**Affiliations:** ^1^ Católica Biomedical Research Centre, Católica Medical School Universidade Católica Portuguesa Lisbon Portugal

**Keywords:** blood–brain barrier, cerebral disease, neuropathology, parasitic infection, vascular dysfunction

## Abstract

The brain vasculature is a critical barrier to maintain central nervous system (CNS) homeostasis. Parasitic infections can profoundly disrupt the brain vasculature, with consequences ranging from subtle neurological alterations to severe, life‐threatening pathologies. In this review, we explore the diverse mechanisms by which endoparasites interact with, modulate and breach CNS blood and lymphatic vessels. We highlight how these pathogens manipulate endothelial function, alter barrier permeability and exploit vascular surface molecules to access or influence the brain. These interactions often trigger local inflammation, endothelial activation and blood–brain barrier breakdown, with significant implications for parasite survival and host pathology. Here, we review the molecular and cellular mechanisms underlying these processes, providing an integrative view of parasite‐vascular crosstalk in the brain and identifying emerging research areas. Understanding these interactions offers new insights into brain vascular disease pathogenesis and may inform future strategies for intervention.

## Graphical abstract

Parasites can affect the blood vessels of the brain, often causing serious neurological problems. This review explains how different parasites interact with and disrupt these vessels, what this means for brain health, and why these processes matter. Understanding these mechanisms may help us develop better ways to prevent or treat brain infections in the future.

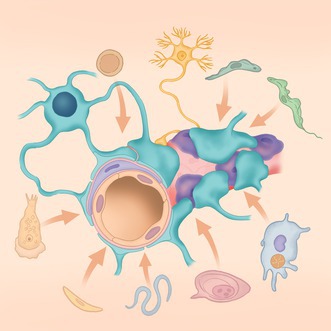

## Abbreviations

ABC (transporter), ATP‐binding cassette

Ang, angiopoietin

AQP4, aquaporin 4

Aβ, amyloid‐beta

BBB, blood–brain barrier

Cav‐1, caveolin 1

CCE, cerebral cystic echinococcosis

CLDN5, claudin 5

CNS, central nervous system

CP, cysteine peptidase

CSF, cerebrospinal fluid

CXCL, C‐X‐C motif chemokine ligand

DC, dendritic cell

EC, endothelial cell

ECM, extracellular matrix

FGF, fibroblast growth factor

GAE, granulomatous amoebic encephalitis

GLUT1, glucose transporter type 1

GPR, G‐protein‐coupled receptor

HRPII, histidine‐rich protein II

IFN, interferon

iRBC, infected red blood cell

JAK, janus kinase

LAT1, L‐type amino acid transporter 1

LRP, Low‐density lipoprotein‐related receptors

LYVE‐1, lymphatic vessel endothelial hyaluronan receptor 1

MEG, major egg glycoprotein

MMP‐9, matrix metalloproteinase‐9

MSA, major serologic antigen

NFκB, nuclear factor kappa‐light‐chain‐enhancer of activated B cells

NVU, neurovascular unit

PDGFB, platelet‐derived growth factor subunit B

PDGFRβ, platelet‐derived growth factor receptor beta

PROX1, prospero‐related homeobox 1

PVLAP, plasmalemma vesicle‐associated protein

Shh, sonic hedgehog signalling

STAT, signal transducer and activator of transcription

TIMP‐1, tissue inhibitor of metalloproteinase 1

TLR, toll‐like receptor

TNF, tumour necrosis factor

TS, transialidase

VEGF, vascular endothelial growth factor

VEGFR, vascular endothelial growth factor receptor

ZO‐1,2,3, zonula occludens 1,2,3

Parasites exhibit diverse traits that enable survival, replication and transmission within their hosts. While many parasites colonise multiple tissues, causing different organ‐specific pathologies [1, 2], infection of the central nervous system (CNS), especially in the brain, causes severe, debilitating and life‐threatening consequences [3]. From a parasite's perspective, colonisation of the CNS poses significant challenges as it possesses remarkable anatomical barriers. Yet, several parasites, including protozoa, helminths and even some ectoparasites, can persist in the brain through mechanisms that incidentally allow them to overcome or bypass CNS barriers, even if cerebral involvement does not directly contribute to their transmission [4]. The breach of the brain's defence mechanisms can precipitate a spectrum of neurological and psychiatric symptoms, which vary depending on the parasite, the host's immune status and the severity of the infection [5]. Common neurological consequences include headaches, seizures, motor disturbances and coma, while psychiatric manifestations may encompass cognitive impairment, mood disorders, personality changes and psychosis [3, 5]. Examples of parasites that affect brain health and their associated clinical signs are described in Table 1. In some cases, clinical manifestations can vary substantially between host species, probably reflecting fundamental differences in brain anatomy, vascular architecture or immune responses. Understanding why and how parasites interact with the CNS is crucial not only for deciphering the pathophysiology of neuroparasitic diseases, but also for identifying novel intervention opportunities to alleviate or prevent clinical symptoms.

**Table 1:** Common neurological and psychiatric manifestations associated with the most common parasitic infections that affect the brain and their host range.

Class	Parasite	Disease	Neurological signs	Psychiatric signs	Host range & frequency of brain disease	Ref
Protozoa	*Plasmodium falciparum*	Cerebral malaria	Headaches, motor and posture disturbances, seizures, coma	Psychosis, delirium, confusion, anxiety	Humans	[6]
	*Babesia* spp.	Cerebral babesiosis	Ataxia, lethargy, anorexia	Confusion, cognitive impairment	Dogs (common), humans (rare), cattle (very rare)	[7]
	*Toxoplasma gondii*	Cerebral toxoplasmosis	Headaches, ataxia, seizures	Anxiety, schizophrenia, epilepsy, psychosis, personality/behavioural changes	Humans, cats (rare), dogs (rare)	[8, 9, 10, 11, 12, 13, 14]
	*Trypanosoma* spp.	Sleeping sickness/(animal) African trypanosomiasis	Anorexia, sensory and circadian disturbances, hemiparesis, tremors, seizures, kinaesthesia, coma	Anxiety, emotional instability, hallucinations, delirium	Humans (common), dogs (common), goats, horses, large ruminants (rare)	[15, 16, 17, 18, 19, 20]
Nematodes	*Loa loa*	*Loa loa* filariasis	Headaches, asthenia, sensory and motor disturbances, coma	Cognitive impairment, parkinsonism	Humans (rare)	[21]
	*Angiostrongylus cantonensis*	Angiostrongyliasis	Headaches, neck stiffness, vomiting, hyperesthesia, paraesthesia	Cognitive impairment, depression, anxiety	Humans, mice, marsupials, nonhuman primates	[22, 23]
Trematodes	*Schistosoma japonicum*	Neuroschistosomiasis	Headaches, ataxia, seizures	Depression, anxiety, altered consciousness	Humans, livestock (e.g. pigs, cattle)	[24]
Cestodes	*Echinococcus* spp.	Cerebral cystic echinococcosis (CCE) or hydatid disease	Headaches, vomiting, hemiparesis, seizures, paralysis, visual impairment	Cognitive impairment, depression, anxiety, personality changes, psychosis, dementia	Humans, livestock (e.g. sheep, cattle, horses)	[25]
	*Taenia* spp.	Neurocysticercosis	Seizures, coma	Depression, cognitive dysfunction, dementia, hallucinations, delusion	Humans	[3, 26]
Amoeba	*Naegleria fowleri*	Primary amoebic meningoencephalitis	Headaches, nausea/vomiting, stiff neck, seizures, photophobia, imbalance, coma	Confusion, hallucinations	Humans, nonhuman primates (rare)	[27, 28]

To appreciate CNS's involvement during parasitic infections, it is first necessary to outline the unique anatomical and physiological barriers that normally protect the brain. The brain integrates and controls nearly every bodily function, from basic physiological processes to higher‐order cognitive and emotional regulation. To maintain its delicate homeostasis, the brain is protected against mechanical forces, infectious agents and metabolites. It is first enclosed by the skull, followed by meninges, which are composed of three layers of connective tissue—dura, arachnoid and pia—and finally surrounded by cerebrospinal fluid. The brain is additionally sheltered from circulating factors by organ‐specific blood endothelial cells (ECs), the cells lining the interior of blood vessels; these form the parenchyma, the choroid plexus and the meningeal vessels. Parenchymal blood capillary ECs acquire specialised features that establish a selective blood–brain barrier (BBB). Yet, the BBB is not composed of ECs alone; it is part of the broader neurovascular unit (NVU), which also includes pericytes, astrocytes, which make the glia limitans, microglia and neurons. Postcapillary venules are important sites for leukocyte transmigration, with a perivascular space that allows immune cells to reside in. Invasion into the brain parenchyma is highly controlled by the glia limitans. Together, these elements maintain barrier integrity, regulate immune and metabolic traffic, and mediate communication between the brain and peripheral circulation. These same structures also represent critical interfaces that parasites must cross, modulate or exploit to establish CNS involvement [3, 5]. Parasites can directly disrupt EC barriers by increasing their permeability; they may activate the endothelium, promoting the expression of adhesion molecules that enhance the recruitment of immune cells; or they can disrupt homeostatic communication channels between ECs and their microenvironment, such as pericytes or astrocytes. All these mechanisms promote inflammation and parasite dissemination. Concomitantly, parasite‐driven inflammatory responses can exacerbate neurological damage, either through direct tissue injury or by altering neurotransmitter balance [29].

Here, we review the principal idiosyncrasies of brain vessels during parasitic infections and discuss how we can exploit them to develop new therapeutics.

## The brain vasculature

**Fig. 1:**
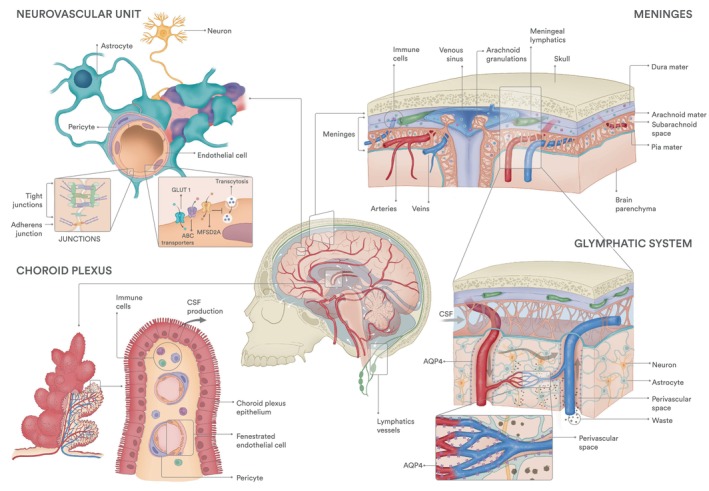
Brain vasculature diversity. Central image—Schematic of a human brain within the skull surrounded by cerebrospinal fluid (CSF, flow pattern is shown by white arrows), showing blood vessels (red) and lymphatic vasculature and lymph nodes (green). Neurovascular Unit—In the brain parenchyma, endothelial capillaries display a blood–brain barrier. These endothelial cells have specialised tight junctions and transporter proteins that control molecular exchanges between blood and neurons. Pericytes, astrocytes and neurons cooperate with endothelial cells to form the neurovascular unit. Choroid plexus—the CSF is produced within the choroid plexus by specialised epithelial cells—choroid plexus epithelium. Choroid plexus's blood vessels are fenestrated and hence more permeable to large molecules and immune cells. Meninges—the dura, arachnoid and pia mater compose the three layers of meninges that rest between the skull and the brain parenchyma. The dura vasculature includes arteries, capillaries, lymphatic vessels and venous sinuses, which act as drainage sites for the brain, skull and scalp. Glymphatic system—CSF enters the brain parenchyma through the spaces around arteries. This fluid is mixed with the interstitial fluid and waste solutes in the parenchyma, and the resulting CSF‐interstitial fluid enters the perivascular spaces around the veins and is drained mostly back to dural sinuses. This exchange of fluid is facilitated by astrocytic AQP4. Zoomed in area shows parenchyma blood capillaries also surrounded by astrocytic AQP4 but lacking perivascular spaces. Grey arrows indicate the CSF and CSF‐ISF flow, and dark grey dots indicate waste solutes that exit the parenchyma.

Despite comprising 2% of body weight, the brain consumes 20% of the body's energy [30], relying on glucose and ketone bodies, but holding no effective storage mechanism or local source. To meet these particular metabolic demands, while remaining protected from exogenous agents and immune cells, the brain has a dense, highly specialised vascular network that allows rapid access to circulating nutrients and oxygen [31]. The BBB is formed by parenchymal capillaries (Fig. 1), complemented by specialised blood vessels in the choroid plexus and meninges. These blood vessels are further surrounded by the perivascular glia limitans, a specialised barrier primarily composed of astrocyte endfeet and specialised basal lamina. Meningeal lymphatic and glymphatic structures support waste clearance. Recent mapping of transcriptional variation in ECs from different vascular beds has deepened our understanding of angiodiversity and organ‐specific molecular signatures [32, 33]. These insights are crucial for identifying the molecular determinants underlying functional differences across distinct vascular beds. Here, we provide an overview of the brain vascular networks and regulatory mechanisms. For a more detailed description, we refer the reader to these excellent reviews [31, 34].

### Parenchyma—The BBB and the neurovascular unit

The BBB controls the passage of substances and cells to maintain brain homeostasis. BBB dysfunction is associated with numerous neurological conditions, such as cancer, dementia, epilepsy, infections, multiple sclerosis and trauma. BBB integrity depends on capillary ECs with specialised features: expression of tight junctions, reduced transcytosis, expression of selective transporters and low leukocyte adhesion molecule expression [31, 34], restricting permeability to small molecules (< 400 Da) [35]. These EC functions are further supported by pericytes, perivascular fibroblasts, glial cells and neurons. This multicellular cluster is called the NVU, which collectively regulates BBB development and maintenance.

#### Junction proteins

The initial recognition that brain ECs were able to hinder the passage of molecules from circulation into the parenchyma came from experiments using injection of tracers done over 50 years ago [36]. At present, it is known that ECs are connected to each other through specific tight, adherens and gap junction proteins. The main classes of tight junction proteins are occludins, claudins and junctional adhesion molecules (JAMs), which are linked to F‐actin cytoskeleton via multi‐domain scaffolding proteins of the peripheral membrane‐associated guanylate kinase family: zonula occludens (ZO‐1, ZO‐2 and ZO‐3) [31, 34]. Occludin (encoded by *Ocln*) is an integral membrane protein localised exclusively to tight junctions [37], highly expressed in the CNS ECs. Yet, *Ocln* ^−/−^ mice develop normal tight junctions [38, 39]. CNS's endothelium also expresses claudin‐5 (*Cldn5*) at high levels [40]. *Cldn5*‐deficient mice showed normal vascularisation of the brain, yet the BBB was compromised for small molecules (< 800 Da), and knock‐out mice died shortly after birth [41]. Brain ECs also hold adherens junctions formed by homotypic binding of endothelial‐specific cadherin, VE‐cadherin, which is linked to the actin cytoskeleton via an α‐ and β‐catenin complex on its cytoplasmic domain [42]. VE‐cadherin is generally required for blood vessel development and integrity and, thus, modulates BBB function [42].

#### Transport proteins

CNS ECs express specialised solute carrier‐mediated transporters, such as glucose transporter type 1 (GLUT1 encoded by *Slc2a1*) and large neutral amino acid transporter 1 (LAT1 encoded by *Slc7a5*) to meet metabolic demands. *Slc2a1*‐deficient animals die before birth, while heterozygous mice replicate GLUT1 deficiency symptoms in humans, including impaired motor activity, microencephaly and increased propensity to seizures [43]. Endothelial‐specific deletion of *Slc7a5* disrupts amino acid balance and causes neurological defects, reversible by branched‐chain amino acid intracerebroventricular administration [44]. In addition, BBB ECs express several ATP‐binding cassette (ABC) transporters that actively pump out toxins and metabolites, contributing to low permeability and resistance to therapeutic agents [31, 34].

#### Transcytosis

Another way for the transport of proteins and molecules across the endothelium is transcytosis. Transcytosis can occur either through ligand‐receptor binding or by nonselective mechanisms in which molecules and plasma membrane interact. At the BBB, the main studied routes are clathrin‐mediated and caveolae‐mediated endocytosis [45], involving clathrin‐coated pockets or caveolin‐ and cavin‐dependent plasma membrane invaginations, respectively [45]. These processes are largely inhibited in BBB ECs by MFSD2a, a lipid transporter for docosahexaenoic acid [46]. Its genetic ablation in mice causes BBB defects, which lead to abnormal cognitive function, neuronal loss and microcephaly [46, 47]. Clathrin‐mediated transcytosis also contributes to amyloid‐β (Aβ) clearance [48]. Pathogens can exploit these pathways: for example, group B *Streptococcus*, which causes bacterial meningitis, increases micropinocytosis, an actin‐dependent transcytosis mechanism, enhancing bacterial invasion [49]. Although transcytosis is tightly regulated to maintain BBB function, the exact contributions of each mechanism in both physiological and pathological conditions remain unclear. Whereas previously BBB physical properties were thought to be limited to capillaries, recent studies showed that postcapillary venules also express some level of specific transporters and efflux pumps, encoded by *Mfsd2a* and *Slc16a1* [50, 51].

#### Auxiliary NVU cells

The NVU encompasses several cell types besides ECs. Pericytes, mural cells that enwrap capillary vessels on their abluminal side, have their highest coverage ratio in the brain vessels [52, 53]. They interact closely with ECs via basement membrane sharing direct contact points that enable metabolite and molecular exchange [31]. Pericytes are recruited by ECs through platelet‐derived growth factor receptor beta (PDGFRβ)–platelet‐derived growth factor subunit B (PDGFB) signalling and contribute to vessel stability via angiopoietin (Ang) 1–TIE2 signalling [54]. They regulate BBB permeability by enhancing EC junction proteins, secreting basement membrane components [55, 56], phagocytising toxic molecules [57] and inducing astrocyte polarisation [55].

Astrocytes, located at the outer NVU layer, form the perivascular glia limitans, supporting barrier integrity by secreting basement membrane proteins [58] and activating the endothelial sonic hedgehog (Shh) signalling pathway. This promotes the expression of tight junction proteins, reduces pro‐inflammatory chemokines and leukocyte adhesion molecules expression, limiting the CNS immune response [59]. In capillaries, the glia limitans is in direct contact with pericytes and ECs, while in postcapillary venules and larger vessels, the glia limitans is not in direct contact with blood vessels, creating a perivascular space (Fig. 1).

#### Molecular mechanisms regulating BBB development

BBB development is a tightly regulated process that starts during embryogenesis. Around embryonic day 10 (E10), ECs from the perineural vascular plexus invade the developing neural tube, guided and attracted by vascular endothelial growth factor (VEGF) A produced by neuroectoderm cells. These early vessels are leaky and acquire BBB properties only by E15 [47, 60]. Wnt signalling is a key regulator of CNS vascular and BBB development [31, 34]. Canonical Wnt ligands (Wnt7a/b), expressed by astroglia, oligodendrocytes and neurons [61, 62, 63], signal through EC‐expressed frizzled receptors [63] and coreceptors [Low‐density lipoprotein‐related receptors (Lrp) 5/6, G‐protein‐coupled receptor (GPR) 124 and Reck] [64]. Activation of canonical Wnt signalling induces β‐catenin‐dependent transcriptional activity, which regulates BBB features, including transporter expression (GLUT1 [65]), tight junction proteins (CLDN5 [66, 67]) and downregulation of transcytosis‐related genes (plasmalemma vesicle‐associated protein (PLVAP) [67]).

### Choroid plexus—Blood–cerebrospinal fluid barrier

The blood–cerebrospinal fluid (CSF) barrier, located in the choroid plexus, separates blood from CSF, which is produced by specialised epithelial cells in each brain ventricle [68]. CSF flows through the subarachnoid spaces around the brain and spinal cord, providing mechanical protection, homeostatic regulation of substances in the brain and facilitating waste removal [68, 69, 70]. Choroid plexus ECs exhibit a distinct transcriptomic profile compared with other CNS ECs, with high expression of *Esm1* [32, 71] and *Plvap* [32]. Their fenestrated structure allows passage of molecules up to 70 kDa, water and other solutes essential for CSF production (Fig. 1) [72]. These ECs associate with pericytes and fibroblasts, but immune, glial and neuronal cells can also be found in this region (Fig. 1) [73]. For many substances and leukocytes, the blood‐CSF barrier is the primary entry point into the brain. Interestingly, it has been shown that the vascular barrier in the brain choroid plexus can be modulated by intestinal inflammation, as bacteria‐derived lipopolysaccharide induces barrier closure via Wnt/β‐catenin signalling pathway [72]. In contrast to BBB development and maintenance, the mechanisms regulating these specialised choroidal ECs remain largely unknown.

### Meninges—Dura, arachnoid, pia and lymphatic vessels

The meninges possess specialised vessel networks that provide and collect blood going into the brain parenchyma. These membranes are an active site of clearance of waste products, such as metabolites, cellular debris and misfolded proteins originating from the brain's activity, and an active site of immune surveillance.

#### Dura, arachnoid, pia

The meninges are composed of three layers: dura, arachnoid and pia mater. The dura mater is the outermost layer, positioned between the skull periosteum and the arachnoid. Its outer surface comprises a thick fibroblast layer embedded within a dense extracellular matrix (ECM), whereas the interface with the arachnoid has a sparser ECM [74, 75]. These layers are tightly connected, except where they split to accommodate dural venous sinuses, which drain the brain, skull and scalp (Fig. 1). The dura houses large blood vessels, including the venous sinuses, veins and arteries that are highly innervated [75], capillaries, lymphatic vessels and resident immune cells.

The arachnoid consists of different fibroblasts organised in layers forming the ceiling of the subarachnoid space, which houses the CSF and is transversed by trabeculae connecting to the pia. In humans, but not mice, these trabeculae also attach to blood vessels crossing the arachnoid, possibly providing structural support [74]. Within the arachnoid, we can also find projections of arachnoid matter into the dural venous sinuses called arachnoid granulations (Fig. 1). These structures allow for the CSF to drain back into the venous blood, although their exact role is still under debate [74].

Finally, the pia mater is a thin fibroblast monolayer intimately wrapping the brain and medulla, being in contact with the astrocyte coat that separates the pia from the parenchyma itself (Fig. 1). This layer holds many fenestrations [74, 75] that allow the passage of larger molecules. The vasculature within the subarachnoid space and pia consists of veins and arteries, bringing blood from and to the brain, but unlike the dura, there are no blood capillaries [74]. Interestingly, meningeal vessels are surrounded by fibroblasts that may constitute unique barrier properties, with a function comparable to astrocytes in the NVU [74].

#### Lymphatics

Over the past 10 years, the existence of meningeal lymphatic vessels, as well as their ability to drain contents from the CSF, has been (re)demonstrated using modern imaging techniques and specific lymphatic markers both in mice [76, 77, 78] and humans [79]. A dural lymphatic network of small capillary‐like vessels lies adjacent to the transverse and superior sagittal sinuses [76, 78] (Fig. 1). These vessels express classical capillary lymphatic EC (LEC) markers, including vascular endothelial growth factor receptor (VEGFR) 3, prospero homeobox protein 1 (PROX1), podoplanin and lymphatic vessel endothelial hyaluronan receptor 1 (LYVE‐1) [76, 78]. Unlike peripheral lymphatics, meningeal lymphatics develop postnatally [80], but remain VEGFR3‐dependent via VEGF‐C secreted by vascular smooth muscle cells, which may explain their peri‐arterial and perivenous preferential location [80, 81]. Dysfunction of meningeal lymphatics has been associated with several diseases, including neurodegenerative Alzheimer's and Parkinson's disease, stroke, ageing, tumour growth or traumatic brain injury [68], due to impaired drainage of CSF, metabolic proteins (ex: Aβ, tau and α‐synuclein proteins), pathogens and immune cells [68].

#### Glymphatics

The brain parenchyma lacks conventional lymphatic vessels, so the waste clearance task is managed by the glymphatic system, a functional analogue of the lymphatic system that relies on glial cells, particularly astrocytes. This system comprises spaces surrounding arteries and veins within the brain parenchyma, allowing solute clearance from CSF and interstitial fluid [82]. CSF flows through the subarachnoid spaces to peri‐arterial regions and is directed into the brain parenchyma, a process facilitated by the presence of astrocytic endfeet expressing aquaporin‐4 (AQP4). The CSF eventually reaches the perivenous space, mixing with interstitial fluid that carries metabolic waste, and is ultimately drained to dural sinuses, meningeal lymphatics and the deep cervical lymph nodes (Fig. 1) [82, 83]. The mechanisms regulating glymphatic network development and function remain poorly understood.

#### Immune surveillance

The brain was long considered an immune‐privileged organ, protected from infection and inflammatory responses. This belief was strengthened by the presence of the BBB and the presumed absence of lymphatic vessels for draining CNS‐derived antigens to lymph nodes [84]. However, we now know that several neuroimmune interactions occur, especially at the meninges and the choroid plexus. Dural and choroid plexus blood vessels are fenestrated, express high levels of PLVAP and adhesion molecules VCAM‐1, ICAM‐1 and P‐selectin, and low levels of CLDN5 and occludin, facilitating immune cell entry from the blood into CSF‐filled spaces [84]. Parenchyma postcapillary venules are preferential sites of immune cell infiltration, and they can be designated as a neuroimmunological BBB. At these vessels, glial cells modulate EC expression of adhesion molecules, such as VCAM‐1, ICAM‐1 or P‐glycoprotein [51, 85, 86], promoting immune cell recruitment and crossing into the perivascular space, thus contributing to immune cell surveillance of the brain [87]. Immune cells can then enter the meningeal lymphatic vessels and be drained out of the brain into the draining lymph nodes. The identity of these cells in the brain ranges from different types of macrophages, especially microglia, neutrophils, T cells, B cells, dendritic cells (DCs) and natural killer cells [88, 89, 90]. Interestingly, one study has discovered that the skull's bone marrow serves as a reservoir for myeloid cells that, upon CNS damage, can migrate into the brain parenchyma through vessels connecting the skull's bone marrow vasculature to the dural veins [91, 92, 93].

## Mechanisms of parasite‐vascular interactions and dysfunction

**Fig. 2:**
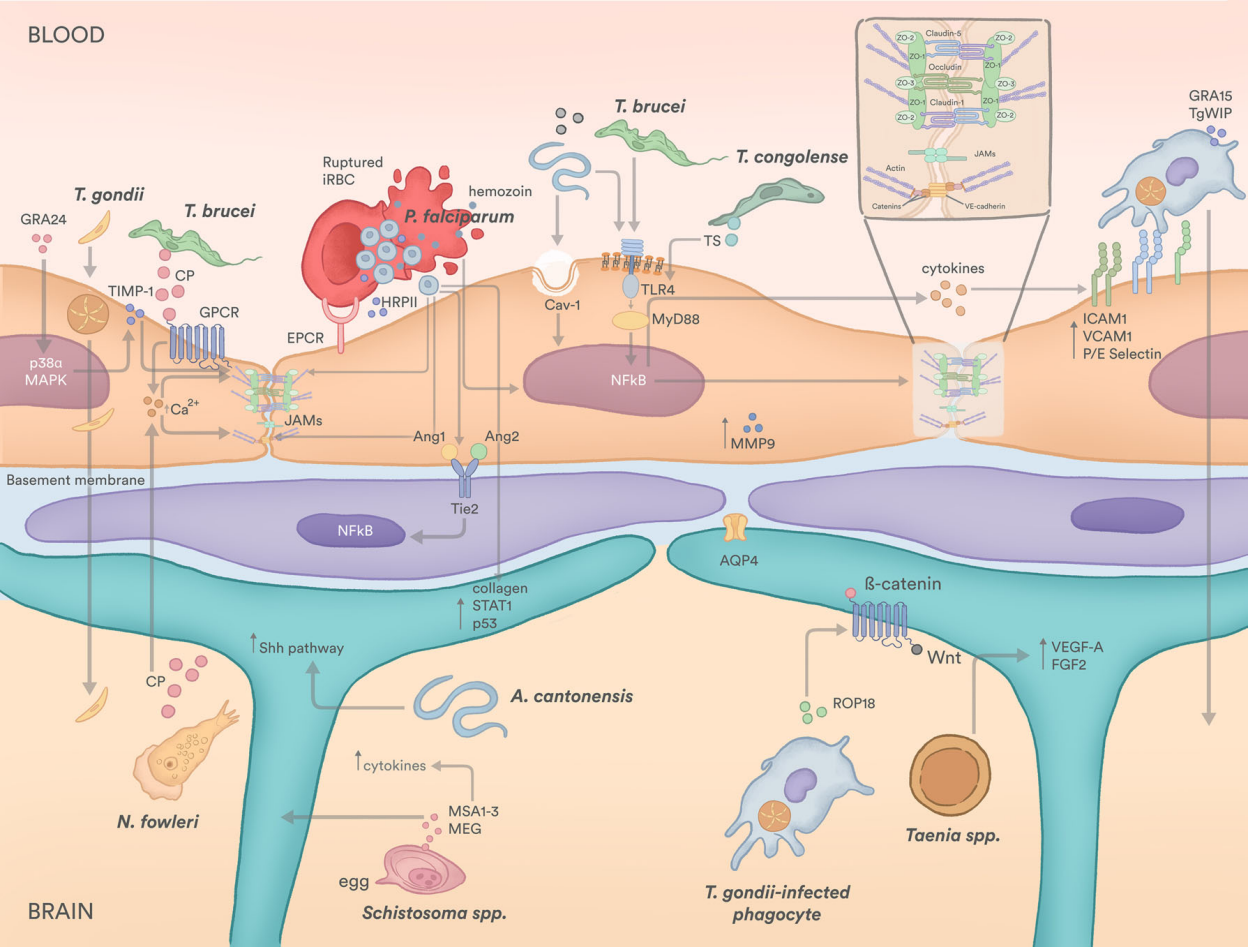
Mechanisms of parasite‐driven blood–brain barrier (BBB) modulation and central nervous system (CNS) invasion. In the bloodstream, *Toxoplasma gondii* secretes GRA24 to activate host p38α mitogen‐activated protein kinase (MAPK) signalling, which triggers tight junction destabilisation via tissue inhibitor of metalloproteinases (TIMP) 1. *T. gondii* can also infect endothelial cells, replicate and egress into the brain parenchyma as an extracellular form. Alternatively, *T. gondii* can infect phagocytes, which traverse the BBB, leading to brain parenchyma colonisation and astrocyte polarisation via ROP18‐induced activation of the Wnt/β‐catenin pathway. Red blood cells infected with *Plasmodium falciparum* (iRBC) cytoadhere to endothelial receptors, such as EPCR. Eventually, they burst to release extracellular parasites and their egress products, including hemozoin and HRPII. These products destabilise tight and adherens junctions, disrupt the angiopoietin (Ang)‐tie axis in pericytes and the transcriptional regulation of collagen, signal transducer and activator of transcription (STAT) 1 and p53 in astrocytes. *Trypanosoma brucei* and *Naegleria fowleri* secrete cysteine peptidases (CP) that lead to increased intracellular calcium, which destabilises both tight and adherens junctions. *T. brucei*, *T. congolense* and *Angiostrongylus cantonensis* can also activate the NF‐κB pathway in the endothelial cells, triggering tight junction destabilisation and cytokine secretion. This can be done via caveolin (Cav) 1, toll‐like receptor (TLR) 4 activation or parasite transialidase (TS) secretion. Additionally, *A. cantonensis* in the brain parenchyma can activate the sonic hedgehog (Shh) pathway in astrocytes. Still in the brain parenchyma, *Schistosoma* spp. eggs release major serological antigen (MSA) 1–3 and major egg glycoprotein (MEG), which act on astrocytes and lead to increased cytokine secretion. *Taenia* spp. also affect astrocytes, but by inducing transcription of vascular endothelial growth factor (VEGF) A and fibroblast growth factor (FGF) 2.

Several parasites can reach and persist in the brain using mechanisms that alter vascular and barrier function. Whether these processes provide an evolutionary advantage to the parasite or represent a pathological by‐product of infection remains debated. Moreover, the impact of brain involvement on pathology can differ between host species. Some parasites cross the BBB to colonise neuronal tissue; others remain adhered to the luminal side while disrupting barrier function and/or elicit host responses; others circumvent the BBB completely. While these strategies differ, they converge on disturbing junctional protein integrity and inducing immune recruitment. Here, we describe three main routes or processes by which parasites hijack the brain vasculature: (i) junction disruption and barrier leakage, (ii) endothelial activation and adhesion, and (iii) BBB circumvention. Often, these processes are interconnected, involve other adjacent cells and invariably result in immune‐mediated amplification of vascular damage. Indeed, a growing body of evidence highlights that host immune responses, while essential for controlling infection, can paradoxically further fuel vascular dysfunction [94] (Fig. 2).

### To damage or not to damage—Junctional breakdown and barrier permeability

Several parasites disturb EC‐EC junctions to facilitate BBB disruption. Parasites that cause African trypanosomiasis provide a good example. *T. brucei* secretes lysosomal cathepsin L‐like cysteine proteases (TbCatL; also known as brucipain) that act on GPCRs called protease activated receptors (PARs) present on brain ECs, activating calcium‐dependent intracellular signalling pathways. The increase in intracellular calcium concentration activates actin cytoskeleton remodelling, which consequently leads to barrier dysfunction and parasite crossing [95, 96]. Despite the direct role of brucipain in BBB breakdown, pro‐inflammatory cytokines, such as interferon‐gamma (IFN‐γ), tumour necrosis factor‐alpha (TNF‐α) and type I interferons (IFN‐α/β), also contribute to BBB permeability and enable both parasite and T‐cell entry into the brain parenchyma [97, 98].

Despite the evolutionary distance between the two species, *T. brucei* and *N. fowleri* share similar mechanisms of BBB disruption. While *N. fowleri* enters the brain through the olfactory nerve, not requiring BBB crossing, it causes BBB breakdown by dysregulation of tight junction proteins CLDN5, occludin and ZO‐1 and actin cytoskeleton disruption. Incubation of brain ECs with either *N. fowleri* or conditioned media decreases barrier integrity [99, 100]. Like in sleeping sickness, secretion of amoebic cysteine proteases may mediate BBB breakdown [101, 102, 103].

A similar strategy is used by *P. falciparum*. As for the previous parasites, soluble factors are the key mediators disrupting EC junctions. In cerebral malaria, BBB breakdown is caused by parasite‐derived molecules that are released upon lysis of infected red blood cells (iRBC‐egress products). Perfusion of microfluidic BBB models with iRBC‐egress media resulted in downregulation of CLDN5, ZO‐1 and VE‐cadherin in ECs [104], as well as reduced Ang‐1 and PDGFRβ in pericytes [104, 105]. This leads to altered junction morphology (with more intercellular gaps) and BBB leakage [104, 105]. These effects appear to be parasite stage‐dependent because perfusion with egress products from RBCs infected with *P. falciparum* trophozoites, which do not cause RBC lysis, has a milder effect on BBB disruption [106]. *P. falciparum*‐derived molecules may also activate β‐catenin‐mediated transcriptional signalling, leading to tight junction disruption [107]. It remains to be established how a signalling pathway that promotes BBB maturation is hijacked to destabilise BBB‐associated EC junctional properties. Other proteins, such as hemozoin, histones and histidine‐rich protein II (HRPII), have been identified as potential mediators of endothelial barrier breakdown during cerebral malaria, acting via multiple pathways to promote inflammation. Hemozoin activates ECs and increases the secretion of matrix metalloproteinase‐9 (MMP‐9), a type IV collagenase, by ECs [108, 109, 110], which leads to tight junction disruption [110]. It has been proposed that histones activate the TLR2 signalling pathway, which in turn activates Src family kinases and p38 MAPK, resulting in a pro‐inflammatory response [111, 112], while HRPII has been proposed to trigger inflammasome formation, where caspase‐1‐dependent signalling activates the nuclear factor kappa‐light‐chain‐enhancer of activated B cells (NF‐κB)/MyD88 pathway [113, 114].

Dysregulation of Wnt/β‐catenin signalling pathway is observed in other parasitic infections: *T. gondii*, for example, impairs neuron and astrocyte differentiation from neural stem cells by disrupting the Wnt/β‐catenin pathway through the effector protein ROP18 [115, 116]. These effects on cell differentiation are important in pathology because they can contribute to birth defects through infection‐related foetal brain tissue abnormalities. *T. gondii* also affects neuron and astrocyte's potential to control EC permeability—for instance, conditioned media from infected neuron and astrocyte cultures impaired the ability of noninfected glial cells to induce ZO‐1 expression and increase transcellular electrical resistance in ECs [117].

*A. cantonensis* uses a different mechanism of junction disruption to breach the BBB. Infection by this parasite leads to an upregulation of caveolin‐1 (CAV‐1) expression in brain ECs, which promotes degradation of occludin and CLDN5 via the NF‐κB signalling pathway [118]. Yet, it is unclear whether this effect is also mediated by secreted molecules or proteins or via direct parasite‐EC interactions.

While BBB breakdown during *T. congolense*‐induced cerebral animal African trypanosomiasis has not been directly quantified, infected mice display haemorrhagic lesions and increased permeability throughout multiple brain regions, which is consistent with immune‐mediated vascular dysfunction [119]. In *T. brucei*‐induced animal African trypanosomiasis, the prevailing view was that BBB traversal by lymphocytes was a prerequisite for parasite entry [98]. This view has since been challenged: parasites were found in the parenchyma of the corpus callosum of RAG1^−/−^ mice at 20 days postinfection [98]; intravital microscopy analysis of infected mice revealed the presence of *T. b. brucei* and *T. b. rhodesiense* in the brain parenchyma at the first day postinfection before microvascular inflammation is detectable [120]; and parasite DNA was found in brain tissue of infected mice 3 days before T cells were observed [121]. So, while T cells facilitate *T. brucei* BBB crossing, it appears that the parasite has developed T‐cell‐independent mechanisms to enter the brain. For example, *T. brucei* colonises the circumventricular organs, choroid plexus and meninges [122, 123, 124, 125], which serve as a point of entry to the brain by providing access to the subarachnoid space and subsequently the pial surface of the brain [126].

Unlike the previous examples, *T. gondii* infects, replicates and exits brain ECs, particularly in the capillaries, to invade the brain, often resulting in isolated events of EC lysis that do not cause haemorrhages [127]. In immunocompetent patients, parasites may also transmigrate without disturbing barrier integrity or permeability, except for foci of parasite hotspots [128, 129]. *T. gondii* secretes GRA24 [130], an effector protein translocated via MYR [131], which induces EC‐derived tissue inhibitor of matrix metalloproteinase (TIMP)‐1 secretion [128] by triggering prolonged autophosphorylation and nuclear translocation of p38α MAP kinase [130]. On the one hand, increased TIMP‐1 protein levels enhance parasite transmigration *in vitro* with minimal barrier disruption [128] and have been detected in brain tissue of infected mice [129, 132]. On the other hand, its genetic deletion results in lower parasite load in the brain [133]. In contrast, in immunocompromised patients, oedema is often observed, both during active episodes of neurological manifestations and due to brain calcification, suggestive of severe disruption of the BBB [134, 135, 136].

### All's well that adheres well—Endothelial activation and vascular adhesion

Parasites can activate ECs, that is induce a pro‐inflammatory EC state, by physical interaction, contact with parasite‐secreted molecules or a combination of both. Parasite‐driven EC activation stimulates the expression of adhesion molecules on its surface, which facilitates parasite sequestration; leukocyte adhesion and transmigration; and secretion of molecules that modulate the immune response. Examples of parasite‐driven EC activation include *T. congolense* [119], *P. falciparum* [106, 137], *T. gondii* [118, 129, 138] and *N. fowleri* [99, 100] infections. All these are characterised by increased ICAM‐1 and VCAM‐1 expression, although the mechanisms involved might differ. EC activation by *T. congolense* is direct: co‐culture with murine lung and bone marrow ECs *in vitro* leads to increased expression of ICAM‐1 and VCAM‐1 through the secretion of parasite trans‐sialidases that activate the NF‐κB pathway, a trigger of inflammatory signals, including IL‐1β, IL‐6 and nitric oxide [139]. *In vivo*, increased expression of ICAM‐1, PECAM‐1 and P/E‐selectin in the brain endothelium is also observed [140]. Furthermore, ICAM‐1 expression is associated with higher parasite sequestration and exacerbated pathology through the facilitation of CD4^+^ T‐cell infiltration into the brain parenchyma, promoting inflammation and neurological impairment [119]. Interestingly, different African trypanosome species have been shown to induce expression of different EC adhesion molecules in the brain [140], suggesting species‐specific mechanisms of BBB modulation. In *T. brucei* infections, the TLR–MyD88 signalling pathway, activated during infection, drives the expression of IFN‐α/β and TNF‐α, which in turn stimulate ECs to express adhesion molecules, for example ICAM‐1 [97, 98]. IFN‐γ produced by T cells, together with IFN‐α/β, stimulates CXCL‐10 secretion by astrocytes, facilitating T‐cell adhesion and diapedesis [98, 141].

*N. fowleri* and *T. gondii* also directly induce the expression of VCAM‐1 and ICAM‐1 on brain ECs [99, 129], despite the molecular mechanisms being less clear. In *N. fowleri* infection, incubation of human brain ECs with the amoeba or its extracellular vesicles leads to robust secretion of IL‐1β, IL‐6, IL‐8 and TNF‐α, but not IL‐10 (an anti‐inflammatory cytokine). This unchecked inflammatory activation of endothelial and glial cells contributes to rapid BBB breakdown and neuronal damage. In cerebral toxoplasmosis, there is an alternative brain invasion mechanism that relies on the upregulation of ICAM‐1. *T. gondii* parasites can be transported across the BBB inside immune cells, in a ‘Trojan horse’ mechanism that is facilitated by the upregulation of ICAM‐1 in infected ECs, promoting leukocyte binding and subsequent transmigration [142, 143]. ICAM‐1/CD18‐mediated sequestration of parasitised mononuclear phagocytes to the brain cortical capillaries enhances brain colonisation in two ways: *T. gondii* tachyzoites egress from infected phagocytes (such as DCs) and either cross the endothelium without replicating or invade ECs, replicate and egress into the brain parenchyma [144]. Both scenarios expedite neuronal colonisation. Importantly, systemic microvascular inflammation (including the increase in ICAM‐1, VCAM‐1 and ELAM‐1) and parasite secretion of effector proteins TgWIP and GRA15 exacerbate phagocyte sequestration [144]. In contrast, *P. falciparum* co‐culture with human brain microvascular ECs does not alter expression of surface markers of endothelial activation, suggesting that these parasites induce EC activation indirectly. Cerebral malaria results in elevated circulating TNF‐α levels, leading to higher ICAM‐1 and VCAM‐1 expression [106, 137], which promotes parasite sequestration and leukocyte adhesion because both iRBCs and T cells bind the brain endothelium via ICAM‐1 [145, 146, 147]. ICAM‐1 upregulation is thought to be mediated through the NF‐κB pathway [148], as observed in *T. congolense* infection [139]. High levels of circulating IL‐1β, IL‐6 and IL‐8 secreted by ECs [149] and recruited immune cells [150] further amplify immune cell recruitment and infiltration [150]. The accumulation of these cells worsens vascular congestion and ischaemia, illustrating how the immune defence can directly contribute to pathology.

Infection with *A. cantonensis* leads to dose‐dependent recruitment of eosinophils into the CSF [151]. These cells degranulate in response to parasite presence, releasing toxic molecules, such as eosinophil peroxidase, major basic protein and cationic proteins. These granule proteins are cytotoxic to both parasites and host tissues, leading to BBB disruption, perivascular inflammation and neurological symptomatology [152]. A role for eosinophil‐derived proteins has been reported in parasitic diseases that result in granulomas, that is organised and compact structures of immune cells, often engulfing parasite cells or products, such as schistosomiasis, neurocysticercosis and granulomatous amoebic encephalitis. For example, in schistosomiasis, adult worms residing in the mesenteric or pelvic veins release eggs that normally transit into the gut or bladder lumen for excretion. However, some eggs become trapped in host tissues and are carried via the circulation to ectopic sites, including the central nervous system. In such cases, granulomas form around these lodged eggs, initiated by the release of parasite antigens [major serologic antigens (MSA)1–3 and major egg glycoprotein (MEG)]. The granulomatous response progresses from predominantly Th1 inflammation to a longer Th2 cytokine response, accompanied by eosinophil, neutrophil and CD4^+^ T‐cell recruitment [153]. The damage extends beyond the granuloma into neighbouring tissue, as postmortem studies show vascular damage, astrocyte activation and demyelination foci [154]. This stage is followed by the productive stage, where the egg is no longer surrounded by a necrotic zone. Instead, the remaining part of the egg is surrounded by lymphocytes and plasmocytes, alleviating astrocyte activation [154]. As healing continues, the egg and lymphocytes become surrounded by fibrotic tissue (in acute infection only; in chronic infection, fibrillary astrogliosis is observed [154]). Patients with schistosomiasis often show vascular lesions, such as necrotising arteritis in small brain arteries, which derive from either the direct interaction of the egg with the vasculature or the deposition of immunocomplexes. These lesions can cause fatal cerebral haemorrhages [155].

Overall, across endoparasitic infections, EC activation emerges as a conserved mechanism of BBB manipulation for facilitating immune cell infiltration at the cost of BBB integrity; though, in many cases, the parasite molecules involved remain poorly defined.

## Not always a bloody affair—The emerging role of lymphatics and glymphatics

Lymphatic involvement in brain modulation during parasitic diseases is an emerging frontier. The discovery of functional meningeal lymphatic vessels in the CNS [78] has reshaped our understanding of immune surveillance and interstitial fluid drainage in the brain. The lymphatic‐CSF interface might be critical for both pathogen clearance and the pathogenesis of brain colonisation.

Lymphatic filariasis (*Wuchereria* sp. and *Brugia* sp.) is the most well‐characterised parasitic disease affecting the lymphatic system. Although it does not involve the CNS, it offers a valuable model of how parasites disrupt lymphatic architecture and physiology. Filarial parasite invasion of the lymphatics leads to vessel dilation, obstruction and valve dysfunction [156]. Additionally, parasite‐secreted immunomodulatory molecules exacerbate inflammation, drive lymphoedema and promote lymphatic fibrosis. While such fibrotic changes have not been documented in brain lymphatics, chronic toxoplasmosis causes meningeal thickening and diffuse astrogliosis [157], which may be shaped, at least partially, by impaired lymphatic clearance. In turn, meningeal lymphatic drainage has been shown to support adaptive immunity against *T. gondii* in the brain by enhancing CD4^+^ and CD8^+^ T‐cell responses through the activation of type 1 and type 2 conventional DCs in the dura mater [158]. However, these lymphatic‐associated responses are insufficient to control the parasite load in the brain, suggesting that effective T‐cell priming may predominantly occur at extracranial sites.

During chronic toxoplasmosis, CSF outflow is reduced, though the role of meningeal lymphatic dysfunction in the development of brain oedema remains unclear. In other neurological conditions, surgical disruption of cervical lymphatic outflow exacerbates cerebral oedema [159, 160], while VEGF‐C‐driven lymphangiogenesis has been shown to reduce brain swelling following traumatic injury [161]. However, in toxoplasmosis, VEGF‐C administration restores CSF drainage to cervical lymph nodes without resolving cerebral oedema [162], indicating that impaired lymphatic drainage is only one component of the broader pathological process. Furthermore, during acute disease, *T. gondii* can enter the brain via the choroid plexus [163].

African trypanosomes also display extensive lymphotropic behaviour. *T. brucei* has long been known to colonise the lymphatic system [164, 165, 166]. Parasites have been detected in brain‐associated lymph nodes, including the cranial deep cervical [167, 168] and mediastinal nodes [168]. Moreover, lymphatic parasite burden fluctuates independently of the blood parasitaemia, supporting the idea that the lymphatics serve as an alternative reservoir that may favour persistence and replication [168, 169]. This view is further corroborated by the observation that the blocking of lymphatic endothelial marker LYVE‐1 improves mouse survival and reduces parasitaemia [168].

Likewise, *N. fowleri* avoids the BBB, instead entering the brain via the cribriform plate and olfactory bulb [170], areas rich in glymphatic influx. While direct studies of glymphatic and lymphatic involvement in primary amoebic meningoencephalitis are lacking, it is plausible that these draining systems facilitate parasite dispersion along perivascular spaces. Conversely, their disruption might exacerbate neurotoxicity by impairing clearance of inflammatory and cytotoxic molecules. In fact, glymphatic dysfunction is associated with oedema and astrocyte reactivity [171], both hallmarks of primary amoebic meningoencephalitis. A mechanistic contributor could be AQP4 mislocalisation at astrocyte endfeet, a feature reported in experimental cerebral malaria [172], despite no significant changes in protein levels [173]. In experimental models of cerebral malaria, brain swelling results from vasogenic oedema rather than perivascular CSF influx pathways. Nonetheless, recovery is contingent on drainage via the basal and nasal submucosa lymphatic networks to the deep cervical lymph nodes as surgical obstruction of the deep cervical lymph nodes undermines resolution [173].

These observations highlight a critical dimension of brain infection: the dual role of glymphatic and lymphatic vasculature as conduits for both parasite dissemination and inflammation resolution. Despite compelling anatomical and experimental evidence pointing to a role for lymphatic and glymphatic systems in the pathophysiology of neurotropic parasitic diseases, their specific contributions remain largely unexplored. A major challenge lies in the methodological limitations of studying them *in vivo* during infection. Moving forward, there is a need for rigorous experimental models that integrate high‐resolution imaging, lymphatic/glymphatic modulation and parasitic infection dynamics. Understanding whether these systems serve primarily as routes of invasion, immune activation hubs, clearance mechanisms or all three could unlock new therapeutic strategies for limiting CNS damage in parasitic diseases.

## Hidden targets—From ECs to the neurovascular unit

Parasites use diverse mechanisms to modulate the brain vasculature, but while strategies vary, the outcome is often similar: increased BBB permeability, immune infiltration and damage to neural tissues. The observation that many neurotropic parasites alter EC behaviour to promote BBB permeability underscores the therapeutic potential of interventions aimed at preserving vascular integrity. Strategies that block parasite adhesion to the endothelium have been discussed in malaria research [174, 175, 176]. However, they are challenging due to the variability and rapid adaptation of the parasite surface to changing environments. Moreover, such strategies would also likely influence immune cell vascular crossing; often, parasites exploit the same host molecules to adhere and cross, potentially compromising adequate immune responses. Alternatively, host‐directed therapies, such as those targeting endothelial protective molecules, including statins or angiopoietin modulators, could be explored to mitigate BBB breakdown and inflammation during parasitic CNS infections [177].

While many efforts have been focused on understanding the impact of parasites on junctional dynamics and disruption, BBB maintenance also relies on transcytosis suppression and on many specific transmembrane transporters. These crucial mechanisms of BBB regulation have been largely neglected in parasite studies, likely caused by inadequate *in vitro* models that mimic these organotypic features. New technologies and methodologies, such as brain microphysiological systems and single‐cell approaches, may lead to important discoveries on the influence of parasites and parasite‐derived molecules in the full spectrum of BBB characteristics.

It has also become clear that endothelial dysfunction is only one facet of the broader neurovascular response to infection. Other cells of the NVU, that is astrocytes and pericytes, as well as microglia and perivascular fibroblasts, have been increasingly recognised as active participants in disease pathogenesis. For example, astrocyte activation is often a hallmark of neurotropic parasitic infections. *A. cantonensis* triggers autophagy in astrocytes via activation of the sonic hedgehog pathway [178], *P. falciparum* induces fibrosis, p53‐mediated apoptosis and Janus kinase‐signal transduction and activation of transcription (JAK–STAT) pathway activation in astrocytes [104], *T. brucei* induces C‐X‐C motif chemokine ligand (CXCL)‐10 secretion by astrocytes in a T‐cell‐mediated manner [141], *T. solium* remodels astrocyte expression profiles [179] and induces astrogliosis [180]; *B. mandrillaris*, the cause of granulomatous amoebic encephalitis (GAE), causes astrocyte activation potentially derived from neuron damage [181]; toxoplasmosis results in astrocyte polarisation to A1 subtype [182, 183]; *N. fowleri*‐induced encephalitis is characterised by astrocyte reactivity [171]. Moreover, *Schistosoma*‐derived granulomas are also associated with astrocyte activation, often sharing characteristics with idiopathic neurodegenerative disease [184]. Despite these findings, the full range of astrocyte responses and their functional consequences remain poorly understood.

Given the high pericyte coverage in brain capillaries and their tight interaction with ECs, parasite‐driven EC activation and BBB crossing likely involve alterations in EC‐pericyte communication and pericyte function. However, a very limited number of studies have explored this axis. Recent work on cerebral malaria showed that Ang‐1 secretion by pericytes decreased after exposure to iRBCs and its egress products, resulting in increased BBB permeability [105], and that endothelial‐pericyte communication pathways, namely PDGF‐PDGFR and Notch signalling and Ang1‐TIE2 axis, are downregulated [104]. Gaining insights in this area might offer an interesting strategy to mitigate brain vascular disease.

## Mind the map—Dissecting brain vascular modulation in space and time

A final frontier in the study of parasitic CNS infections lies in pinpointing the precise temporal, anatomical and cellular sites of parasite invasion, replication and associated host responses. Most studies have relied on endpoint analyses, broad anatomical assessments or bulk ‘omics approaches, masking the dynamics of parasite invasion and host response. Dissecting the precise anatomical niches and progression over time is essential to define critical windows of vulnerability, inform therapeutic timing and understand region‐specific neuropathology.

Emerging technologies are now making this spatial and temporal dissection possible. High‐resolution *in vivo* imaging allows dynamic tracking of parasite behaviour and vascular responses in real‐time. Spatial transcriptomics and proteomics enable mapping of infection‐induced molecular changes at cellular resolution across defined brain regions. Integration with single‐cell technologies can reveal time‐dependent alterations in all cells of the neurovascular unit at once. Microphysiological models, such as organ‐on‐chip or organoid systems, offer new platforms to recapitulate early invasion events, define the sequence of cellular perturbations and test temporal interventions in a controlled setting. Importantly, recent advances in tissue modelling are improving their organotypic fidelity, with vascularised brain organoids providing enhanced structural and functional relevance to the *in vivo* brain environment [185, 186, 187]. Furthermore, the integration of such systems into host–parasite research has begun to demonstrate their potential for dissecting infection dynamics and therapeutic opportunities [104, 188, 189, 190, 191].

While incorporating space as a variable in both animal models and *in vitro* systems can offer further mechanistic insights into how parasites modulate the vascular–brain interface, incorporating time will be key to capturing the kinetics of host–parasite interactions and revealing temporal windows for optimal intervention. Longitudinal sampling in experimental infections, paired with computational approaches to reconstruct trajectories of cellular responses, can help define cause‐and‐effect relationships and tipping points that precede irreversible damage. Ultimately, a space‐and‐time‐resolved understanding of brain vascular modulation will not only refine our mechanistic insights but also sharpen the development of stage‐specific, regionally targeted and temporally optimised interventions.

## Conclusion

In conclusion, parasitic infections of the CNS reveal a complex interplay between pathogens and host cells, in particular ECs. Endothelial dysfunction emerges as a central feature, although growing evidence points to a broader, coordinated disruption of neurovascular homeostasis. To move towards precision therapeutics, a concerted effort to map parasite–host interactions in both spatial and temporal dimensions is critical. For that, integrating emerging imaging, ‘omics and microphysiological systems might allow us to delineate the sequence, location and nature of host responses that drive disease or confer tolerance. Importantly, such approaches will also need to account for interspecies differences in brain and vascular biology, which may explain why neuropathology emerges in some hosts but not in others. This might also help resolve to what extent parasite interactions with brain vessels represent adaptive traits that support parasite success or rather reflect collateral consequences of infection that primarily drive host pathology. Such insights will be essential to understand how parasites disturb the delicate balance of cellular interactions that define a healthy NVU.

## Author contributions

LL and SA‐M wrote the original draft. All authors contributed to writing and editing the final manuscript. SSP and CAF proposed and conceived the study.
